# Statement of Retraction: Toll-like receptor 3 gene regulates cataract-related mechanisms via the Jagged-1/Notch signaling pathway

**DOI:** 10.1080/21655979.2026.2667574

**Published:** 2026-05-21

We, the Editors and Publisher of the journal *Bioengineered*, have retracted the following article from publication.

Xie, W., Yu, Q., Wang, L., Shao, Y., Bo, Q., & Wu, G. (2022). Toll-like receptor 3 gene regulates cataract-related mechanisms via the Jagged-1/Notch signaling pathway. *Bioengineered*, 13(6), 14359–14369. https://doi.org/10.1080/21655979.2022.2085391

Following publication, internal integrity audits revealed concerns around several authorship changes which were made between submission and acceptance of the article.

We have contacted the authors for an explanation, but we have not received a response. As determining authorship and contributions is core to the integrity and accountability of published work, we are therefore retracting the article. The authors have been informed of this decision.

We have been informed in our decision-making by our policy on publishing ethics and integrity and COPE guidelines.

The retracted article will remain online to maintain the scholarly record and will be digitally watermarked on each page as ‘Retracted’.

